# Emergency hernia hospitalizations in older adults with and without multimorbidity

**DOI:** 10.1007/s10029-026-03725-4

**Published:** 2026-05-20

**Authors:** Yangzi Liu, Karole Collier, James Sharpe, Drew Goldberg, Jasmine Hwang, Leslie M. Okorji, Maria S. Altieri, Rachel R. Kelz

**Affiliations:** 1 Department of Surgery, Hospital of the University of Pennsylvania, 3400 Spruce Street, 4 Maloney, Philadelphia, PA 19104 USA; 2 Center for Surgery and Health Economics, Perelman School of Medicine, 3400 Civic Center Boulevard, Philadelphia, PA 19104 USA; 3 Leonard Davis Institute, 3641 Locust Walk #210, Philadelphia, PA 19104 USA

**Keywords:** Emergency surgery, Hernia, Multimorbidity

## Abstract

**Purpose:**

Hernia repairs have differences in outcomes based on hernia type. Information regarding hernia burden in the emergency setting is lacking. Among older adults, who have the greatest prevalence of hernia and the need for emergent repair, little data on the impact of multimorbidity on outcomes exist. We aim to define the burden of emergency hernia on hospitals and to compare outcomes of older adults with and without multimorbidity.

**Methods:**

This was a nationwide retrospective cohort study of Medicare beneficiaries admitted emergently from 2015–2018 with a principal diagnosis of an umbilical, ventral, parastomal, femoral, or inguinal hernia. The primary outcome was all-cause inpatient mortality. Multivariable logistic regression was performed.

**Results:**

Among 47,687 hospitalized patients, there were 4,612 (9.7%) umbilical, 17,707 (37.1%) ventral, 2,486 (5.2%) parastomal, 3,754 (7.9%) femoral, and 15,138 (31.7%) inguinal hernias. Multimorbidity was common (*n* = 24,393, 51.2%). Multimorbid patients had significantly higher rates of inpatient mortality (4.1% vs 1.1%), intensive care needs (48.6% vs 22.7%), discharge to a skilled nursing facility (SNF) (23.9% vs 11.7%), and 30-day readmission (22.0% vs 13.9%) than non-multimorbid patients. After adjustment, multimorbid patients had higher odds of death during index hospitalization (odds ratio = 1.98, CI: 1.53–2.56), intensive care needs (1.89, 1.74–2.06), and discharge to a SNF (1.52, 1.35–1.72) than non-multimorbid patients. Outcomes varied significantly based on hernia type.

**Conclusions:**

We define rates and outcomes of emergency hernia hospitalization in older adults across different hernia types and multimorbidity status. Multimorbid older adults hospitalized for ventral hernias had greatest risk of inpatient death. These data will permit improved patient counseling and shared-decision making for older patients admitted for emergency hernias across hernia types.

**Supplementary Information:**

The online version contains supplementary material available at 10.1007/s10029-026-03725-4.

## Introduction

Hernia repair is one of the most performed surgical procedures in the United States, with over one million performed annually [1, 2]. Inguinal hernias represent the most prevalent hernia type, affecting approximately 15–20% of the general population whereas other hernia types such as femoral hernias or parastomal hernias are less common [3]. Management of hernias differ significantly based on hernia type. For example, despite the high rate of recurrence for parastomal hernia repair (up to 73%), surgical societies provide little guidance on their recommended treatment, resulting in a high surgeon threshold for planned elective repair and differences amongst surgeons [4]. In contrast, guidelines recommend the repair of all femoral hernias at the time of diagnosis due to high rates of complication without surgical treatment [5]. No specific guidelines exist for the management of hernias in the high-risk older adult population.

Although most hernia repairs are performed in an elective fashion, a significant proportion of 9–15% will still be treated in the emergent setting [6–8]. The prevalence of emergent repairs in older adults is increasing over time [6] and increasing age has been found to be an independent risk factor for requiring emergent hernia repair [9]. Older adults who undergo emergent as opposed to elective hernia repairs suffer worse outcomes including increased mortality, reoperation, and readmission [8–10]. In addition to older age, multimorbidity confers greater risk of poor outcomes in patients hospitalized for emergent operations [11, 12].

Despite the prevalence of hernia repairs and their disproportionate impact on older adults when performed emergently, there is a paucity of epidemiological data of hernias in older adults. While established that older adults experience poor outcomes with emergent hernia repair, there lacks specific data on outcomes stratified by hernia type or multimorbidity. We aim to define the burden of emergency hernias on hospital utilization in older adults and to compare the outcomes of those patients with and without multimorbidity, stratified by hernia type.

## Methods

### Population

This is a nationwide, retrospective observational cohort study using Centers for Medicare and Medicaid (CMS) claims data between April 2015 and December 2018. Patients aged ≥ 65.5 years who were admitted from the emergency department with a principal diagnosis code of hernia as outlined by Shafi, et al. between October 2015 and November 2018 were included to allow for a six-month look back and one-month look forward period [13]. Hernia diagnoses were classified as umbilical, ventral (including incisional), parastomal, femoral, inguinal, and other unspecified hernias using ICD-10 codes (Appendix: Supplemental Table 1). Patients with a principal diagnosis code of hiatal hernia were excluded as its treatment and management differs from other hernia types. Patients who did not have complete Medicare Part A and Part B records or received care at a health maintenance organization within six months prior to and after index admission were excluded due to concerns regarding missing data.

### Outcomes

The primary outcome of interest was all-cause mortality during the index hospitalization. Secondary outcomes include intensive care stay, length of stay (LOS), discharge to a skilled nursing facility (SNF), and 30 day readmission rates (calculated from the day of discharge from index hospitalization and excluding patients who died during the time frame). Extended LOS was defined to be a stay greater than the 75th percentile of the cohort.

### Covariates

Multimorbidity was defined as the presence of a Qualifying Comorbidity Set (QCS) as set forth by Silber, et al. [11]. A QCS is a specific grouping of medical conditions that impact risk and has been associated with increased 30 day mortality in general surgery patients [11, 12]. Multimorbidity defined by the presence of QCSs offers a more specific and standardized way to define at-risk surgical patients which aids in clinical decision making and generalizability [12]. Other covariates include age, sex, race/ethnicity, hernia type, dual eligibility status (Medicare and Medicaid) as a proxy for socioeconomic status [14], frailty [15–19], presence of sepsis (calculated using the Angus-Sepsis Score) [20], presence and severity of comorbidities (determined using the Elixhauser Comorbidity Indices) [21], and treatment type. Treatment was defined as operative or non-operative based on the presence (or absence) of CPT or ICD-10 procedure codes for hernia repair during the index hospitalization (Appendix: Supplemental Table 2).

### Statistical analysis

Means and standard deviations (SD) for continuous variables and counts with proportions for categorical variables were computed to define the burden of disease by hernia type among hospitalized older adults. Chi-square tests and generalized Wilcoxon rank sum tests were used to compare characteristics and outcomes of older adults with and without multimorbidity. Subset analyses by hernia type were performed.

Multivariable logistic regression models were used to compare risk-adjusted outcomes of older adults with and without multimorbidity. Multivariable quantile regression estimating median differences was used to compare risk-adjusted length of stay (LOS) between those with and without multimorbidity. Independent models were developed to examine outcomes by each hernia type.

All analyses were done in SAS 9.4 (SAS Institute Inc., Cary, NC, USA) and R 4.4.0 (R Core Team, R Foundation for Statistical Computing, Vienna, Austria), and all statistical tests were considered significant at a two-sided level of significance of 0.05 (α ≤ 0.05), unless otherwise specified. There was no missing data in the final analytic cohort. A Bonferroni adjustment for multiple comparisons was used in the subset analyses.

This research was designed, analyzed, and reported in accordance with the Strengthening the Reporting of Observational Studies in Epidemiology (STROBE) guidelines [22].

## Results

**Fig. 1:**
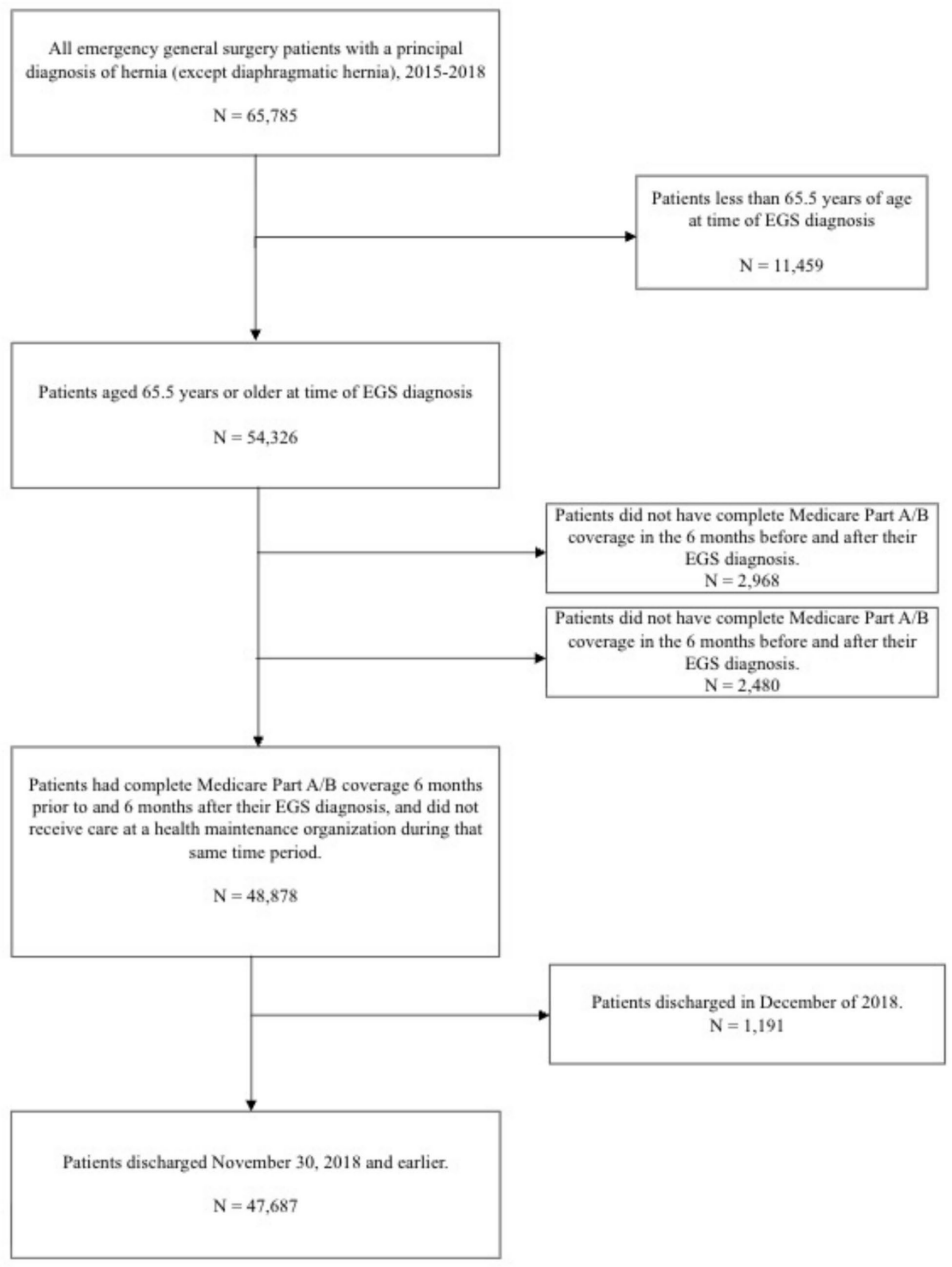
Consort diagram

**Fig. 2:**
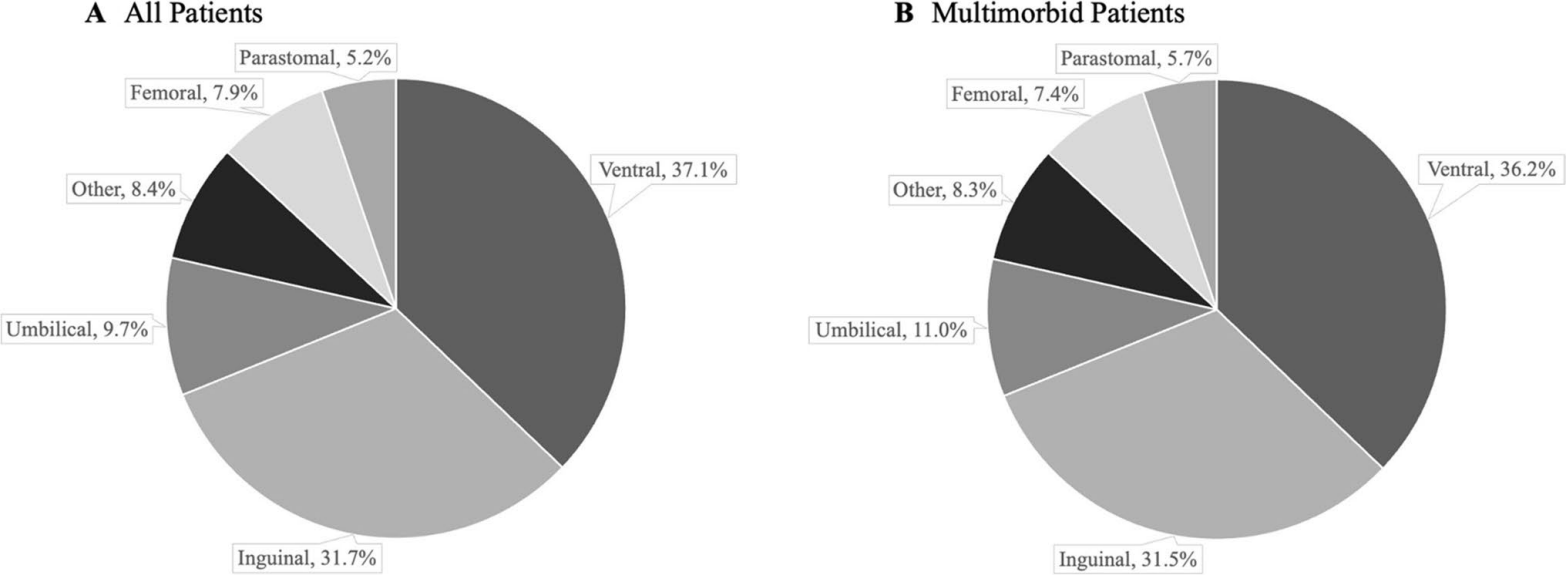
Hospitalization breakdown by hernia type

There were 47,687 older adults included in the study cohort (Fig. 1).The mean ± SD age was 78.9 ± 8.3 years. There was variation in the rates of hospitalization by hernia type with most older adults hospitalized with ventral hernias (*n* = 17,707, 37.1%) or inguinal hernias (*n* = 15,138, 31.7%). Fewer older adults were hospitalized with umbilical hernias (*n* = 4,612, 9.7%), hernias not otherwise specified (*n* = 3,990, 8.4%), femoral hernias (*n* = 3,754, 7.9%), and parastomal hernias (*n* = 2,486, 5.2%) (Fig. 2). Most of the study population underwent operative treatment (*n* = 33,552, 70.4%) and half (*n* = 24,393, 51.2%) were multimorbid.

Older adults with multimorbidity were significantly older (mean age in years ± SD, 80.0 ± 8.4 vs 77.7 ± 8.1, *p* < 0.001), frailer (claims-based frailty index (mean ± SD) 0.18 ± 0.06 vs 0.13 ± 0.03, *p* < 0.001) and had higher rates of sepsis (7.9% vs 1.4%, *p* < 0.001) than non-multimorbid older adults. The distribution of rates of hospitalization by hernia type was similar between multimorbid and non-multimorbid patients (Fig. 2). Multimorbid patients had lower rates of operative management (67.8% vs 73.0%, *p* < 0.001) than non-multimorbid patients (Table 1).

**Table 1:** Patient characteristics by multimorbidity status

Characteristic	Non-multimorbid	Multimorbid	All patients
Total Number, *n* (%)	23,294 (48.8)	24,393 (51.2)	47,687 (100)
Age (years), mean ± SD	77.7 ± 8.1	80.0 ± 8.4	78.9 ± 8.3
Gender, *n* (%)			
Male	9,658 (41.5)	11,776 (48.3)	21,434 (44.9)
Claims-Based Frailty Index[15], mean ± SD	0.13 ± 0.03	0.18 ± 0.06	0.16 ± 0.06
Angus Sepsis[20] (yes), *n* (%)	331 (1.4)	1,935 (7.9)	2,266 (4.8)
Medicaid Dual-Eligible (yes), *n* (%)	2,341 (10.0)	4,210 (17.3)	6,551 (13.7)
Received an Operation, *n* (%)	17,006 (73.0)	16,546 (67.8)	33,552 (70.4)
Hernia Type, *n* (%)			
Umbilical	1,939 (8.3)	2,673 (11.0)	4,612 (9.7)
Ventral	8,877 (38.1)	8,830 (36.2)	17,707 (37.1)
Parastomal	1,092 (4.7)	1,394 (5.7)	2,486 (5.2)
Femoral	1,952 (8.4)	1,802 (7.4)	3,754 (7.9)
Inguinal	7,458 (32.0)	7,680 (31.5)	15,138 (31.7)
Other	1,976 (8.5)	2,014 (8.3)	3,990 (8.4)

All *p*-values < 0.001

*SD* Standard deviation

Among all older adults hospitalized for a hernia, 2.6% (*n* = 1,257) died during the index hospitalization, 35.9% (*n* = 17,138) received care in the intensive care unit (ICU), 18.0% (*n* = 8,577) were discharged to a SNF, and 18.1% (*n* = 8,618) were readmitted within 30 days. Mean LOS (days ± SD) was 5.9 days ± 6.1.

Older adults with multimorbidity experienced higher rates of inpatient mortality (4.1% vs 1.1%, *p* < 0.01) and rates differed by hernia type (Table 2). When stratified by age (65–74, 75–84, 85 +), rates of inpatient mortality increased with increasing age (Appendix: Supplemental Table 3). This pattern was consistent across all hernia types and independent of multimorbidity status.

**Table 2:** Unadjusted outcomes of emergency hernia patients by multimorbidity status

Outcome by hernia type	Non-multimorbid	Multimorbid	All patients
**Umbilical**	***N* = 1,939**	***N* = 2,673**	***N* = 4,612**
Mortality during index hospitalization, *n* (%)	14 (0.7)	88 (3.2)	102 (2.1)
ICU care, *n* (%)	428 (22.1)	1,243 (46.5)	1,671 (36.2)
Discharged to a skilled nursing facility, *n* (%)	195 (10.1)	499 (18.7)	694 (15.0)
LOS index hospitalization (calendar days), mean ± SD	4.4 ± 4.1	5.9 ± 5.4	5.3 ± 4.9
Readmissions within 30 days, *n* (%)^	244 (12.7)	573 (22.2)	817 (18.1)
**Ventral**	***N* = 8,877**	***N* = 8,830**	***N* = 17,707**
Mortality during index hospitalization, *n* (%)	83 (0.9)	362 (4.1)	445 (2.5)
ICU care, *n* (%)	2,085 (23.5)	4,404 (49.9)	6,489 (36.6)
Discharged to a skilled nursing facility, *n* (%)	1,000 (11.3)	2,057 (23.3)	3,057 (17.3)
LOS index hospitalization (calendar days), mean ± SD	5.1 ± 4.1	7.2 ± 6.6	6.1 ± 5.6
Readmissions within 30 days, *n* (%)	1,291 (14.7)	2,064 (24.4)	3,355 (19.4)
**Parastomal**	***N* = 1,092**	***N* = 1,394**	***N* = 2,486**
Mortality during index hospitalization, *n* (%)	17 (1.6)	73 (5.2)	90 (3.6)
ICU care, *n* (%)	284 (26.0)	708 (50.8)	992 (39.9)
Discharged to a skilled nursing facility, *n* (%)	112 (10.3)	317 (22.7)	429 (17.3)
LOS index hospitalization (calendar days), mean ± SD	6.0 ± 9.0	8.0 ± 7.9	7.1 ± 8.4
Readmissions within 30 days, *n* (%)	224 (20.8)	376 (28.5)	600 (25.0)
**Femoral**	***N* = 1,952**	***N* = 1,802**	***N* = 3,754**
Mortality during index hospitalization, *n* (%)	22 (1.1)	83 (4.6)	105 (2.8)
ICU care, *n* (%)	393 (20.1)	896 (49.7)	1,289 (34.3)
Discharged to a skilled nursing facility, n (%)	259 (13.3)	504 (28.0)	763 (20.3)
LOS index hospitalization (calendar days), mean ± SD	4.6 ± 4.7	7.0 ± 5.9	5.7 ± 5.4
Readmissions within 30 days, *n* (%)	229 (11.9)	338 (19.7)	567 (15.5)
**Inguinal**	***N* = 7,458**	***N* = 7,680**	***N* = 15,138**
Mortality during index hospitalization, *n* (%)	78 (1.0)	285 (3.7)	363 (2.4)
ICU care, *n* (%)	1,478 (19.8)	3,405 (44.3)	4,883 (32.3)
Discharged to a skilled nursing facility, *n* (%)	905 (12.1)	1,870 (24.3)	2,775 (18.3)
LOS index hospitalization (calendar days), mean ± SD	4.0 ± 5.7	5.9 ± 7.0	4.9 ± 6.5
Readmissions within 30 days, *n* (%)	973 (13.2)	1,622 (21.9)	2,595 (17.6)
**Other**	***N* = 1,976**	***N* = 2,014**	***N* = 3,990**
Mortality during index hospitalization, *n* (%)	31 (1.6)	121 (6.0)	152 (3.8)
ICU care, *n* (%)	615 (31.1)	1,199 (59.5)	1,814 (45.5)
Discharged to a skilled nursing facility, *n* (%)	266 (13.5)	593 (29.4)	859 (21.5)
LOS index hospitalization (calendar days), mean ± SD	6.8 ± 4.9	9.4 ± 6.8	8.1 ± 6.1
Readmissions within 30 days, *n* (%)	279 (14.3)	405 (21.4)	684 (17.8)
**All hernias**	***N* = 23,294**	***N* = 24,393**	***N* = 47,687**
Mortality during index hospitalization, *n* (%)	245 (1.1)	1,012 (4.1)	1,257 (2.6)
ICU care, *n* (%)	5,283 (22.7)	11,855 (48.6)	17,138 (35.9)
Discharged to a skilled nursing facility, *n* (%)	2,737 (11.7)	5,840 (23.9)	8,577 (18.0)
LOS index hospitalization (calendar days), mean ± SD	4.8 ± 5.2	6.9 ± 6.8	5.9 ± 6.1
Readmissions within 30 days, *n* (%)	3,240 (13.9)	5,378 (22.0)	8,618 (18.1)

All *p*-values < 0.001

^ % denominator excludes patients who died

*ICU* Intensive care unit; *LOS* Length of stay

Older adults with multimorbidity also experienced higher rates of ICU care (48.6% vs 22.7%, *p* < 0.01) and discharge to a SNF (23.9% vs 11.7%, *p* < 0.01) when compared to the non-multimorbid (Table 2). Mean LOS for older adults with multimorbidity was 2.1 days longer than the non-multimorbid (6.9 days ± 6.8 vs 4.8 ± 5.2; *p* < 0.01). Multimorbid patients had significantly higher rates of 30-day readmission (22.0%) when compared to non-multimorbid patients (13.9%) (*p* < 0.01).

Unadjusted outcomes stratified by hernia type followed patterns seen by all patients; multimorbid patients had higher rates of worse outcomes and longer LOS than non-multimorbid patients (Table 2). Specific rates of various outcomes differed by hernia type. For example, inpatient mortality ranged from 0.7% (umbilical) to 1.6% (parastomal) for non-multimorbid patients and 3.2% (umbilical) to 6.0% (other) for multimorbid patients. ICU rates ranged from 19.8% (inguinal) to 31.1% (other) for non-multimorbid patients and 44.3% (inguinal) to 59.5% (other) for multimorbid patients. Rates of SNF discharge ranged from 10.1% (umbilical) to 13.5% (other) for non-multimorbid patients and 18.7% (umbilical) to 29.4% (other) for multimorbid patients. Rates of readmission within 30 days of discharge ranged from 11.9% (femoral) to 20.8% (parastomal) for non-multimorbid patients and 19.7% (femoral) and 28.5% (parastomal) for multimorbid patients.

LOS also differed by hernia type. Excluding other hernias not otherwise specified, multimorbid older adults hospitalized for parastomal hernias had the longest LOS (8.0 ± 7.9) while those hospitalized for inguinal (5.9 ± 7.0) and umbilical (5.9 ± 5.4) hernias had the shortest LOS. Similarly, non-multimorbid older adults hospitalized for parastomal hernias had the longest LOS (6.0 ± 9.0) while older adults with inguinal hernias had the shortest LOS (4.0 ± 5.7).

After adjustment for potential confounding, multimorbid patients had approximately twice the odds of death during their index hospitalization compared to non-multimorbid patients (OR = 1.98, CI = 1.53 – 2.56). Additionally, multimorbid patients had 89% higher odds of receiving ICU care (1.89, 1.74 – 2.06) and 52% higher odds of being discharged to a SNF (1.52, 1.35 – 1.72) than non-multimorbid patients. After adjusting for potential confounders, multimorbid patients had similar odds of prolonged length of stay (1.12, 1.00 – 1.24) and readmission within 30 days (0.95, 0.87 – 1.04) when compared to non-multimorbid patients (Table 3).

**Table 3:** Adjusted outcomes of emergency hernia patients by multimorbidity status

Outcome by hernia type	Odds ratio (95% CI), ref = non-Multimorbid	*p*-value
**Umbilical**		
Mortality during index hospitalization	–	
ICU care	1.82 (1.36 – 2.43)	< 0.0001*
Discharged to a skilled nursing facility	1.98 (1.29 – 3.03)	0.0016*
Prolonged LOS index hospitalization^	1.14 (0.76 – 1.70)	0.5206
Readmissions within 30 days	0.93 (0.70 – 1.24)	0.6134
**Ventral**		
Mortality during index hospitalization	1.86 (1.22 – 2.83)	0.0040*
ICU care	1.87 (1.62 – 2.16)	< 0.0001*
Discharged to a skilled nursing facility	1.75 (1.41 – 2.16)	< 0.0001*
Prolonged LOS index hospitalization^	1.16 (0.96 – 1.41)	0.1202
Readmissions within 30 days	0.96 (0.84 – 1.11)	0.5755
**Parastomal**		
Mortality during index hospitalization	3.74 (1.34 – 10.39)	0.0120
ICU care	2.17 (0.74 – 6.37)	0.1565
Discharged to a skilled nursing facility	0.57 (0.20 – 1.65)	0.3013
Prolonged LOS index hospitalization^	2.67 (0.55 – 13.04)	0.2252
Readmissions within 30 days	0.74 (0.30 – 1.78)	0.4974
**Femoral**		
Mortality during index hospitalization	2.07 (1.56 – 3.71)	0.0140
ICU care	2.15 (1.77 – 2.61)	< 0.0001*
Discharged to a skilled nursing facility	1.38 (1.06 – 1.79)	0.0154
Prolonged LOS index hospitalization^	1.26 (0.95 – 1.69)	0.1127
Readmissions within 30 days	0.80 (0.67 – 0.96)	0.0171
**Inguinal**		
Mortality during index hospitalization	1.95 (1.15 – 3.31)	0.0130
ICU care	1.46 (1.07 – 2.00)	0.0165
Discharged to a skilled nursing facility	1.89 (1.12 – 3.19)	0.0176
Prolonged LOS index hospitalization^	0.89 (0.54 – 1.45)	0.6368
Readmissions within 30 days	0.95 (0.70 – 1.30)	0.7499
**Other**		
Mortality during index hospitalization	1.62 (0.93 – 2.82)	0.0900
ICU care	2.02 (1.66 – 2.47)	< 0.0001*
Discharged to a skilled nursing facility	1.52 (1.19 – 1.95)	0.0008*
Prolonged LOS index hospitalization^	1.30 (1.07 – 1.58)	0.0086
Readmissions within 30 days	1.03 (0.80 – 1.31)	0.8411
**All hernias**		
Mortality during index hospitalization	1.98 (1.53 – 2.56)	< 0.0001*
ICU care	1.89 (1.74 – 2.06)	< 0.0001*
Discharged to a skilled nursing facility	1.52 (1.35 – 1.72)	< 0.0001*
Prolonged LOS index hospitalization^	1.12 (1.00 – 1.24)	0.0428
Readmissions within 30 days	0.95 (0.87 – 1.04)	0.2538

– Cell size too low and cannot be reported due to constraints of data use agreement

^Determined to be a stay greater than 75th percentile of cohort

*Bonferroni correction yields significance threshold of 0.0071

*ICU* Intensive care unit; *LOS* Length of stay

All models controlled for potential confounders including: age, claims-based frailty index, dual eligibility, Angus sepsis score, year of EGS admission, sex, race, and all individual Elixhauser comorbidities

When stratified by hernia type, there were differences in adjusted outcomes by multimorbidity status. For example, older multimorbid adults hospitalized with parastomal hernias had similar odds (OR, 95% CI), of all measured outcomes including inpatient mortality (3.74, 1.34 – 10.39), ICU care (2.17, 0.74 – 6.37), and discharge to a SNF (0.57, 0.20 – 1.65) when compared to those without multimorbidity. Meanwhile, patients with ventral hernias had higher odds of inpatient mortality (1.86, 1.22 – 2.83), ICU care (1.87, 1.62 – 2.16), and discharge to a SNF (1.75, 1.41 – 2.16) when compared to those without multimorbidity.

## Discussion

To our knowledge, this is the first nationwide cohort study to characterize emergency hernia hospitalizations among older adults across all abdominal wall hernia types while explicitly evaluating the role of multimorbidity. The overall inpatient all-cause mortality rate was moderate at 2.6% with the oldest patients (age 85 +) experiencing higher rates of mortality than younger patients (65–74, 75–84 years). Approximately 1 in 3 patients needed critical care during their hospitalization and 1 in 5 were discharged to a SNF or readmitted within 30 days. Further, although multimorbid hernia patients experienced higher rates of risk-adjusted inpatient all-cause mortality, ICU utilization, and discharge to a SNF, their risk of prolonged LOS and readmission within 30 days was not significantly elevated.

Prior studies characterizing hernia burden focused on specific hernia types (i.e. only groin hernias [1]) [3] without inclusion of all hernias, were limited to single-center samples [7], and lacked outcome data [6]. Our data adds to the literature on differences in outcomes between multimorbid older adults and non-multimorbid older adults as well as across hernia types to better describe the specific risks related to all emergency hernia repairs.

A prior study of older adult emergency surgery patients that underwent operative treatment for a multitude of conditions showed that multimorbid patients had worse outcomes than non-multimorbid patients including increased inpatient mortality, 180 day mortality, and higher rates of readmission [23]. We show that older multimorbid patients hospitalized specifically for emergency hernias similarly have worse outcomes and lower operative rates than non-multimorbid patients.

These data are helpful for surgeons when counseling patients who are admitted emergently or urgently for a hernia. The large sample size, a strength of this study, allows for more reliable referencing of the results with greater power when discussing expected lengths of hospitalization, likelihood of needing critical care, facility discharge, readmission rates, and death for such patients with and without multimorbidity. Older patients face challenging decisions when it comes to healthcare as their values impact care impacting quantity vs quality of life [24]. Patients interviewed after surgery note experiences with not being adequately informed of post-operative challenges and having their surgical informational needs unmet which contributed to difficult recovery after surgery [25]. Our results are instrumental in addressing such needs unique to the older surgical population and in turn improve the way healthcare is delivered to them.

In addition to inpatient counseling, these data can also be applied to outpatient counseling for elective surgical hernia repairs through comparison to data established in literature to guide decision-making. For example, retrospective cohort studies of patients who underwent an elective ventral hernia repair found that older patients (age > 65 years) had readmission rates between 4.2–5.6% depending on frailty and age [26, 27]. Our cohort of emergency ventral hernia patients had 30 day readmission rates of 19.4%. Additionally, the odds of any complication studied (including mortality, ventilator use > 48 h, readmission, and return to the operating room) in Wu, et.al’s retrospective review of inguinal hernia repair patients were significantly increased in emergent as compared to elective surgery for all age groups, and most pronounced in patients > 80 years of age [28]. Further research is needed comparing outcomes for multimorbid patients that undergo elective as compared to emergent hernia repairs as such data could better inform more specific decision making for earlier hernia intervention in a multimorbid cohort.

The strength in this study lies in its generalizability with its large sample size reflecting nationwide patterns and inclusion of all hernia types. All claims-based research is limited based on provider accuracy of diagnosis coding which varies between providers and institutions. Specific data regarding hernia size is lacking. A small minority of patients included in this study did not have a defined hernia type and was diagnosed as “other, undefined” hernia. However, the cohort of this study was very large and likely able to account for low rates of misclassification. As with all observational studies, this study was limited by the potential for unmeasured residual confounding.

## Conclusion

This retrospective cohort analysis of older patients hospitalized emergently or urgently for a hernia describes patterns of hospitalization and outcomes by hernia type and multimorbidity status. Ventral and inguinal hernias are the most common hernia types requiring admission for this patient population. Multimorbid patients had higher odds of worse outcomes as compared to non-multimorbid patients including inpatient mortality, needing critical care, and discharge to a SNF. These data can be used by general surgeons when counseling patients and making treatment-decisions regarding emergency hernias in older patients.

## Supplementary Information

Below is the link to the electronic supplementary material.

Supplementary file1 (DOCX 22 KB)
