# Supplementary material for: Emergency hernia hospitalizations in older adults with and without multimorbidity
Source: Hernia. 2026 May 20;30(1):224. doi: 10.1007/s10029-026-03725-4 (PMC13190803; doi:10.1007/s10029-026-03725-4)
Supplement: Supplementary file 1 — Supplementary file1 (DOCX 22 KB) [file 10029_2026_3725_MOESM1_ESM.docx]

**Emergency Hernia Hospitalizations in Older Adults With and Without Multimorbidity**

Supplementary Appendix

| **eTable 1: Billing Codes Used to Determine Hernia Type** | |
| --- | --- |
| **Hernia Type** | **ICD-10 Codes** |
| Umbilical | “K420” “K421” “K429” |
| Incisional/Ventral | “K430” “K431” “K432” “K436” “K437” “K439” |
| Parastomal | “K433” “K434” “K435” |
| Femoral | “K4120” “K4121” “K4190” “K4191” “K4100” “K4101” “K4110” “K4111” “K4130” “K4131” “K4140” “K4141” |
| Inguinal | “K4000” “K4001” “K4010” “K4011” “K4020” “K4021” “K4030” “K4031” “K4040” “K4041” “K4090” “K4091” |
| Other | “K435” “K436” “K437” “K450” “K451” “K458” “K460” “K461” “K469” |

| **eTable 2: Procedure Codes Used to Determine Operative Status** | | |
| --- | --- | --- |
| **Code Type** | **Description** | **Codes** |
| CPT | Laparoscopic hernia repair | "49652" "49653" "49654" "49655" "49656" "49657"; |
| CPT | Open hernia repair | "49560" "49561" "49565" "49566" "49570" "49572" "49585" "49587" "49505" "49591" "49592" "49593" "49594" "49595" "49596" "49613" "49614" |
| ICD-10-PCS | Open Abdominal Wall Repair | "0WQF0ZZ" "0WQF3ZZ" "0WQF4ZZ" "0WQFXZ2" "0WQFXZZ" |
| CPT | Hernioplasty Open | "49491" "49492" "49493" "49494" "49495" "49496" "49497" "49498" "49499" "49500" "49501" "49502" "49503" "49504" "49505" "49506" "49507" "49508" "49509" "49510" "49511" "49512" "49513" "49514" "49515" "49516" "49517" "49518" "49519" "49520" "49521" "49522" "49523" "49524" "49525" "49526" "49527" "49528" "49529" "49530" "49531" "49532" "49533" "49534" "49535" "49536" "49537" "49538" "49539" "49540" "49541" "49542" "49543" "49544" "49545" "49546" "49547" "49548" "49549" "49550" "49551" "49552" "49553" "49554" "49555" "49556" "49557" "49558" "49559" "49560" "49561" "49562" "49563" "49564" "49565" "49566" "49567" "49568" "49569" "49570" "49571" "49572" "49573" "49574" "49575" "49576" "49577" "49578" "49579" "49580" "49581" "49582" "49583" "49584" "49585" "49586" "49587" "49588" "49589" "49590" "49591" "49592" "49593" "49594" "49595" "49596" "49597" "49598" "49599" "49600" "49601" "49602" "49603" "49604" "49605" "49606" "49607" "49608" "49609" "49610" "49611" "49612" "49613" "49614" "49615" "49616" "49617" "49618" "49619" "49620" "49621" "49622" "49623" |
| CPT | Hernioplasty Laparoscopic | "49624" "49625" "49626" "49627" "49628" "49629" "49630" "49631" "49632" "49633" "49634" "49635" "49636" "49637" "49638" "49639" "49640" "49641" "49642" "49643" "49644" "49645" "49646" "49647" "49648" "49649" "49650" "49651" "49652" "49653" "49654" "49655" "49656" "49657" "49658" "49659" |
| CPT | Anterior abdominal wall repair | C7565 |

| **eTable 3: Inpatient Mortality by Hernia Type and Age** | | | | | |
| --- | --- | --- | --- | --- | --- |
| **Age** | **Unadjusted Results, (n, %)** | | | **Adjusted Results** | |
|  | **Non-Multimorbid** | **Multimorbid** | **p-value*** | **Odds Ratio (95% CI)**  **ref = Non-Multimorbid** | **p-value** |
| **Umbilical** | | | | | |
| 65-74 | – | 28 (2.5) | <0.001 | 1.86 (0.41 – 8.49) | 0.421 |
| 75-84 | – | 34 (3.4) | <0.001 | 3.00 (0.61 – 14.68) | 0.174 |
| 85+ | – | 26 (4.7) | 0.211 | 0.94 (0.23 – 3.75) | 0.928 |
| **Ventral** | | | | | |
| 65-74 | 24 (0.6) | 107 (3.3) | <0.001 | 2.85 (1.04 – 7.81) | 0.041 |
| 75-84 | 30 (0.9) | 135 (4.1) | <0.001 | 1.77 (0.86 – 3.62) | 0.120 |
| 85+ | 29 (1.9) | 120 (5.3) | <0.001 | 1.64 (0.87 – 3.07) | 0.124 |
| **Parastomal** | | | | | |
| 65-74 | – | 14 (3.3) | 0.030 | 2.81 (0.24 – 32.43) | 0.407 |
| 75-84 | – | 34 (6.3) | <0.001 | 7.31 (1.24 – 42.98) | 0.028 |
| 85+ | – | 25 (5.9) | 0.285 | 3.05 (0.53 – 17.48) | 0.211 |
| **Femoral** | | | | | |
| 65-74 | – | – | – | – | – |
| 75-84 | 11 (1.4) | 27 (4.2) | 0.001 | 0.9 (0.33 – 2.49) | 0.840 |
| 85+ | – | 54 (6.3) | <0.001 | 3.35 (1.47 – 7.65) | 0.004 |
| **Inguinal** | | | | | |
| 65-74 | – | 23 (1.7) | <0.001 | 4.13 (0.28 – 60.28) | 0.300 |
| 75-84 | 23 (0.8) | 78 (2.8) | <0.001 | 1.92 (0.67 – 5.48) | 0.222 |
| 85+ | 46 (1.9) | 184 (5.3) | <0.001 | 1.8 (0.94 – 3.41) | 0.074 |
| **Other** | | | | | |
| 65-74 | – | 14 (3.0) | <0.001 | 1.94 (0.38 – 9.81) | 0.422 |
| 75-84 | – | 30 (4.0) | 0.002 | 1.38 (0.44 – 4.27) | 0.581 |
| 85+ | 18 (3.7) | 77 (9.6) | <0.001 | 1.56 (0.75 – 3.24) | 0.239 |
| **All hernias** | | | | | |
| 65-74 | 45 (0.5) | 188 (2.7) | <0.001 | 2.82 (1.38 – 5.76) | 0.004 |
| 75-84 | 79 (0.9) | 338 (3.7) | <0.001 | 2.05 (1.28 – 3.30) | 0.003 |
| 85+ | 121 (2.2) | 486 (5.8) | <0.001 | 1.71 (1.21 – 2.41) | 0.002 |
| – Cell size too low and cannot be reported due to constraints of data use agreement  *p-values derived from Fisher's exact test comparing index mortality rates between multimorbid and non-multimorbid patients | | | | | |
